# Inhibition of Intercellular Cytosolic Traffic via Gap Junctions Reinforces Lomustine-Induced Toxicity in Glioblastoma Independent of MGMT Promoter Methylation Status

**DOI:** 10.3390/ph14030195

**Published:** 2021-02-27

**Authors:** Matthias Schneider, Anna-Laura Potthoff, Bernd O. Evert, Marius Dicks, Denise Ehrentraut, Andreas Dolf, Elena N. C. Schmidt, Niklas Schäfer, Valeri Borger, Torsten Pietsch, Mike-Andrew Westhoff, Erdem Güresir, Andreas Waha, Hartmut Vatter, Dieter H. Heiland, Patrick Schuss, Ulrich Herrlinger

**Affiliations:** 1 Department of Neurosurgery, University Hospital Bonn, 53127 Bonn, Germany; anna-laura.potthoff@ukbonn.de (A.-L.P.); s4madick@uni-bonn.de (M.D.); denise.ehrentraut@ukbonn.de (D.E.); s4elschm@uni-bonn.de (E.N.C.S.); valeri.borger@ukbonn.de (V.B.); erdem.gueresir@ukbonn.de (E.G.); hartmut.vatter@ukbonn.de (H.V.); patrick.schuss@ukbonn.de (P.S.); 2 Brain Tumor Translational Research Affiliation, University Hospital Bonn, 53127 Bonn, Germany; niklas.schaefer@ukbonn.de (N.S.); awaha@uni-bonn.de (A.W.); ulrich.herrlinger@ukbonn.de (U.H.); 3 Department of Neuropathology, University Hospital Bonn, 53127 Bonn, Germany; torsten.pietsch@ukbonn.de; 4 Department of Neurology, University Hospital Bonn, 53127 Bonn, Germany; b.evert@uni-bonn.de; 5 Institute of Experimental Immunology, University Hospital Bonn, 53127 Bonn, Germany; andreas.dolf@ukbonn.de; 6 Division of Clinical Neurooncology, Department of Neurology, University Hospital Bonn, 53127 Bonn, Germany; 7 Department of Pediatrics and Adolescent Medicine, University Medical Center Ulm, 89075 Ulm, Germany; andrew.westhoff@uniklinik-ulm.de; 8 Translational NeuroOncology Research Group, Medical Center University of Freiburg, 79106 Freiburg, Germany; dieter.henrik.heiland@uniklinik-freiburg.de; 9 Department of Neurosurgery, University of Freiburg, 79106 Freiburg, Germany; 10 Faculty of Medicine, University of Freiburg, 79106 Freiburg, Germany

**Keywords:** glioblastoma, gap junctions, intercellular cytosolic traffic, lomustine, meclofenamate

## Abstract

Glioblastoma is a malignant brain tumor and one of the most lethal cancers in human. Temozolomide constitutes the standard chemotherapeutic agent, but only shows limited efficacy in glioblastoma patients with unmethylated O-6-methylguanine-DNA methyltransferase (MGMT) promoter status. Recently, it has been shown that glioblastoma cells communicate via particular ion-channels—so-called gap junctions. Interestingly, inhibition of these ion channels has been reported to render MGMT promoter-methylated glioblastoma cells more susceptible for a therapy with temozolomide. However, given the percentage of about 65% of glioblastoma patients with an unmethylated MGMT promoter methylation status, this treatment strategy is limited to only a minority of glioblastoma patients. In the present study we show that—in contrast to temozolomide—pharmacological inhibition of intercellular cytosolic traffic via gap junctions reinforces the antitumoral effects of chemotherapeutic agent lomustine, independent of MGMT promoter methylation status. In view of the growing interest of lomustine in glioblastoma first and second line therapy, these findings might provide a clinically-feasible way to profoundly augment chemotherapeutic effects for all glioblastoma patients.

## 1. Introduction

Glioblastoma cells have been shown to assemble to a multicellular communicating network based on ultra-long and interconnecting membrane protrusions—so-called tumor microtubes (TMs) [1,2]. The resulting cell–cell connections are supported by Connexin43 (Cx43)-based gap junctions that mediate intercellular cytosolic exchange and tumor tissue homeostasis [2]. As a consequence, Cx43-knockdown in primary glioblastoma cells reduced communication between astrocytoma cells via intercellular calcium waves and, additionally, diminished the number of glioblastoma cells with TM formation [2]. Functional TMs, in turn, were associated with resistance to cytotoxic treatment through formation of a self-repairing syncytial network, which was markedly disturbed when Cx43 was down-regulated [3].

Previously, we have demonstrated that pharmacological inhibition of gap junctions via INI-0602, a novel experimental gap junction inhibitor, increased the susceptibility of O6-methylguanine-DNA methyltransferase (MGMT) promoter methylated glioblastoma cells to standard chemotherapeutic agent temozolomide (TMZ) [4]. Together with other in vitro studies that suggested gap junction inhibition to render glioblastoma cells more vulnerable to TMZ therapy [5,6,7], these findings raise hope for novel therapeutic implementations. 

Besides TMZ, lomustine as a nitrosurea compound has become a standard chemotherapeutic drug in recurrent glioblastoma [8,9,10]. Furthermore, in a recently published randomized phase 3 clinical trial, additional application of alkylating agent lomustine to TMZ standard therapy was accompanied by a survival benefit for patients with newly-diagnosed MGMT promoter methylated glioblastoma [11]. Based on the abovementioned promising insights into a Cx43-based communicating malignant network, we asked whether a gap junction-targeted therapeutic approach might reinforce not only TMZ-induced, but also lomustine-induced antitumoral effects. Our findings reveal that meclofenamate (MFA), a nonsteroidal anti-inflammatory drug (NSAID) that has been reported to block gap junction-mediated intercellular communication in various physiological cell entities [12,13], profoundly sensitizes glioblastoma cells to lomustine-mediated cell death. In contrast to TMZ, this effect was independent of MGMT promoter methylation status. With regard to MFA as a U.S. Food and Administration (FDA)-approved drug, these findings recommend the idea of a gap junction-targeted therapy to constitute a clinically-feasible way to further render glioblastoma cells susceptible for a lomustine-based chemotherapeutic approach. 

## 2. Results

### 2.1. Characterization of Primary Human Glioblastoma Cell Populations

**Figure 1:**
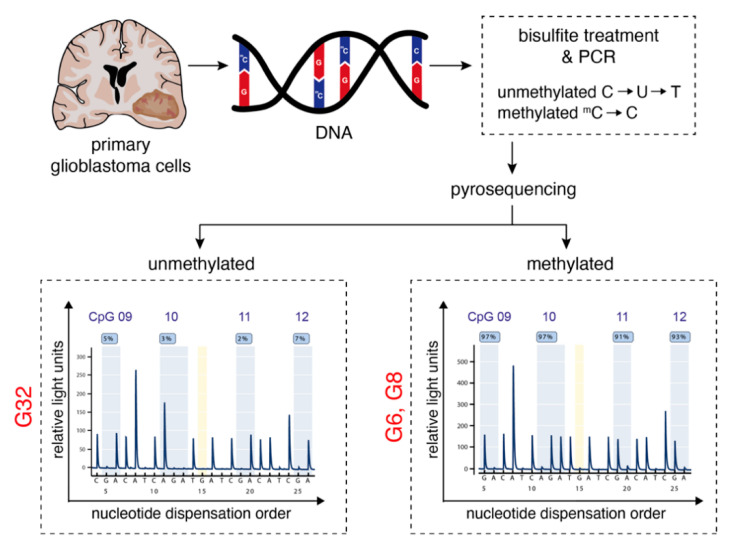
Characterization of human primary glioblastoma cell populations. Workflow illustration of MGMT promoter methylation analysis: Glioblastoma cells were isolated by mechanical disaggregation from surgical specimens obtained from three patients with WHO grade IV astrocytoma. After cell culture was established, DNA was isolated and a pyrosequencing assay was used to determine CpG methylation of the MGMT promoter. The lower panel depicts the methylation level at 4 individual CpG positions for the G32 (left) and G6 (right). Within the program, a guanine signal (G) in this reverse assay indicates the methylated state of the cytosine of the respective CpG dinucleotide, whereas an adenine (A) signal represents the unmethylated state. A, adenine; C, cytosine; CpG, 5′-C-phosphate-G-3′; G, guanine; MGMT, O-6-methylguanine-DNA methyltransferase; T, thymine; U, uracil.

Three primary human glioblastoma cell populations (G6, G8, and G32) deduced from intraoperative surgical specimens were taken for the following experiments. MGMT promoter methylation analysis revealed a significant hypermethylation of the MGMT promoter in the investigated region of the MGMT gene for G6 and G8 cell populations, whereas G32 exhibited an unmethylated MGMT promoter status as determined by pyrosequencing (Figure 1).

### 2.2. Meclofenamate Blocks Gap Junction-Mediated Intercellular Cytosolic Traffic

**Figure 2:**
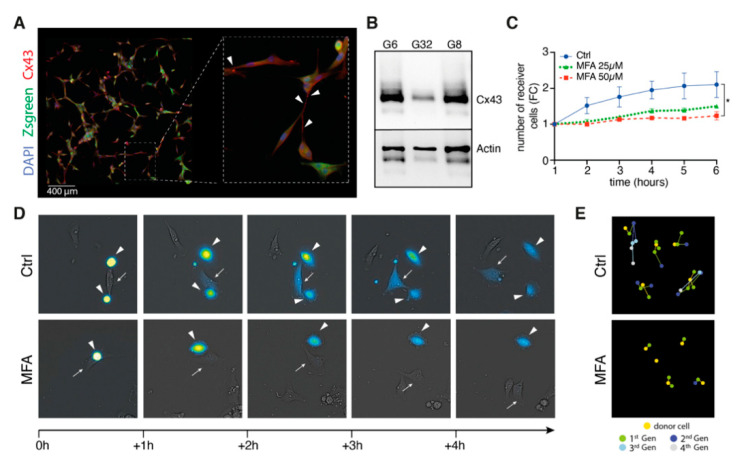
Meclofenamate blocks gap junction-mediated intercellular cytosolic traffic. (**A**) Representative fluorescence image of Cx43 expression for G8 primary glioblastoma cell population. White arrowheads point to Cx43 expression along the membrane protrusions. (**B**) Verification of Cx43 expression using Western Blot analysis. (**C**) Quantification of receiver cells that have incorporated the calcein dye that is transferred through cells connected by gap junctions for the G8 cell population. Data is given as mean ± SEM. *p*-values are determined by Wilcoxon matched-pairs signed rank test. * denotes *p* < 0.05. (**D**) Representative fluorescence images acquired by the IncuCyte^®^ S3 Live-Cell Analysis System illustrating the transfer of the calcein dye from donor cells (arrowheads) to (potential) receiver cells (arrows). (**E**) Graphical illustration of dye spreading from one donor cell to multiple generations of receiver cells. Cx43, connexin-43; FC, fold change; Gen, generation; MFA, meclofenamate.

MFA has been reported to block connexin-43-mediated intercellular communication in various physiological cell entities [12,13]. Therefore, we asked whether drug repurposing of MFA in the context of glioblastoma might sufficiently block intercellular gap junction-mediated calcein dye transfer as a surrogate readout for intercellular cytosolic traffic within preformed TM-based glioblastoma networks. Immunofluorescence imaging revealed syncytial multicellular network morphology for our primary cell culture system (Figure 2A) with positive Cx43 staining alongside characteristic and ultra-long TM formations (Figure 2A,B). Realtime-imaging of fluorescence-guided measurements of calcein cell to cell transfer showed sufficient inhibition of intercellular gap junction-mediated cytosolic traffic in a concentration- and time-dependent manner (Figure 2C–D). While intercellular calcein dye transfer reached up to the fourth generation of receiver cells for untreated control populations, it was dropped at the second generation for 50 µM of MFA treatment (Figure 2E).

### 2.3. Reduced Intercellular Connectivity via Gap Junctions Synergizes to Lomustine-Mediated Antitumoral Effects

**Figure 3:**
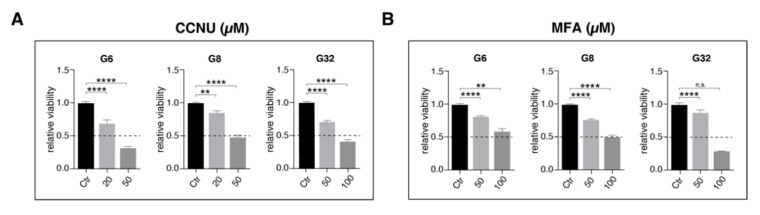
Relative cell viability after treatment for 144 h of the G6, G8, and G32 cell populations with (**A**) CCNU and (**B**) MFA using a MTT assay. Mean ± SEM is depicted. ** and **** denote *p <* 0.01 and *p <* 0.0001. CCNU, lomustine; MFA, meclofenamate.

Our data suggest MFA to significantly reduce syncytial intercellular communication via inhibition of gap junction-mediated intercellular cytosolic traffic. As intercellular connectivity is considered a major hallmark in fostering tumor cells resistant to cytotoxic treatment modalities [14], we next asked whether MFA might harbor the potential of rendering glioblastoma cells more vulnerable to lomustine-mediated antitumoral effects. In order to define effective treatment concentrations for lomustine, metabolic activity was monitored for 144 h after drug application using the MTT assay. A concentration of 20 µM (G6) and 50 µM (G8, G32) was chosen for the following experiments with regard to about a half maximal effect (EC50 value) at 144 h (Figure 3A, Figure S1A). Compared with this, 50 µM of MFA that had been shown to block gap junction-mediated cytosolic cell-to-cell transfer, did not markedly affect cellular viability (Figure 3B, Figure S1B).

Compared with this, 50 µM of MFA that had been shown to block gap junction-mediated cytosolic cell-to-cell transfer, did not markedly affect cellular viability (Figure 3B, Supplementary Figure S1B). 

**Figure 4:**
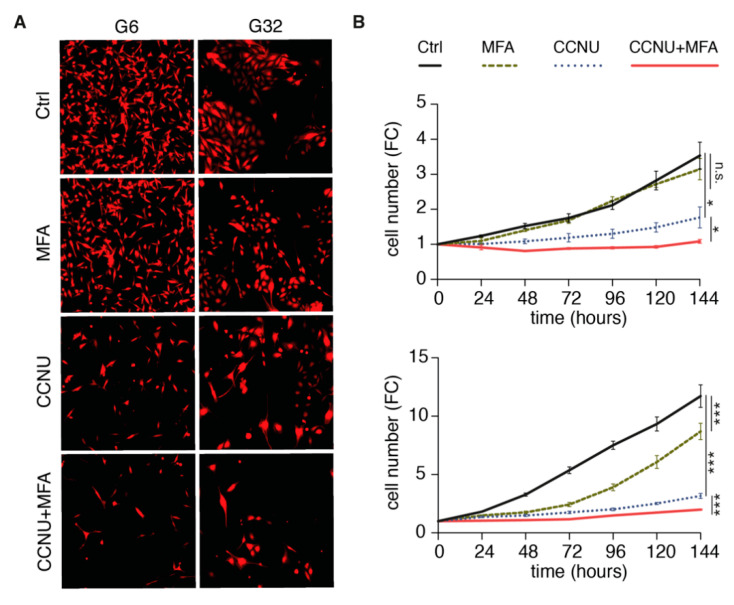
Meclofenamate-induced effects on cell proliferation. (**A**) Representative fluorescence images acquired by the IncuCyte^®^ S3 Live-Cell Analysis System after indicated treatment for 144 h of the G32 (upper panel) and the G6 (lower panel) cell populations. (**B**) Quantification of cell number from fluorescence images obtained in a 24-h interval over a period of 144 h. Mean ± SEM is depicted. * and *** denote *p* < 0.05 and *p* < 0.001. CCNU, lomustine; FC, fold change; MFA, meclofenamate.

Treatment with lomustine revealed a significant reduction in cell number compared to untreated controls (Figure 4A,B). While MFA single treatment yielded a significant decrease only in case of G6, the cell number was further diminished for combination treatment of MFA and lomustine compared to lomustine alone (fold chance values at 144 h for combination versus (vs.) lomustine single treatment: G32: 1.8 vs. 1.1; G6: 3.2 vs. 2).

**Figure 5:**
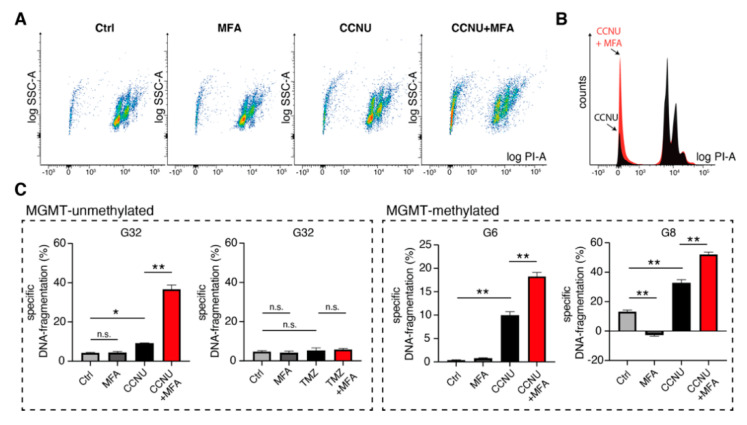
Meclofenamate sensitizes glioblastoma cells to lomustine-mediated cell death. (**A**) Representative scatter plots from flow cytometric analysis of propidium-iodide stained nuclei for G32 cell population for indicated treatment conditions. (**B**) Histogram overlay of flow cytometric analysis for CCNU treatment and combination treatment with MFA. (**C**) Mean ± SEM of specific DNA-fragmentation rates for MGMT-unmethylated (left) and MGMT-methylated (right) cell populations after 144 h of treatment. Treatment was performed with either CCNU, MFA or the combination of both. In case of MGMT-unmethylated cell population G32 cells were additionally treated with TMZ and the combination of TMZ with MFA. * and ** denote *p* < 0.05 and *p* < 0.01. CCNU, lomustine; MFA, meclofenamate; PI, propidium iodide; SSC-A, side scatter area; TMZ, temozolomide.

In order to analyze the underlying mechanisms of observed effects on cell number, specific DNA-fragmentation rates of propidium-iodide stained nuclei were measured as surrogate readout for cell death. MFA single treatment did not show a significant increase in the rate of dead cells compared to controls (Figure 5A–C). For the combination treatment with lomustine, we observed a profound sensitizing effect for lomustine-mediated cell death (Bliss values were 2.8 for G32, 1.7 for G6 and 1.7 for G8). This sensitizing effect was independent from MGMT-promotor methylation status: While TMZ single and TMZ-MFA combination treatment did not lead to elevated levels of dead cells in case of MGMT-promotor unmethylated G32 cell populations, lomustine showed significant effects for both single and combination treatment (Figure 5C).

### 2.4. Meclofenamate-Mediated Sensitizing Effects are Accompanied by Reduced Aurora Kinase A Expression and Reveal Elevated Programmed Cell Death Pathway Signaling

**Figure 6:**
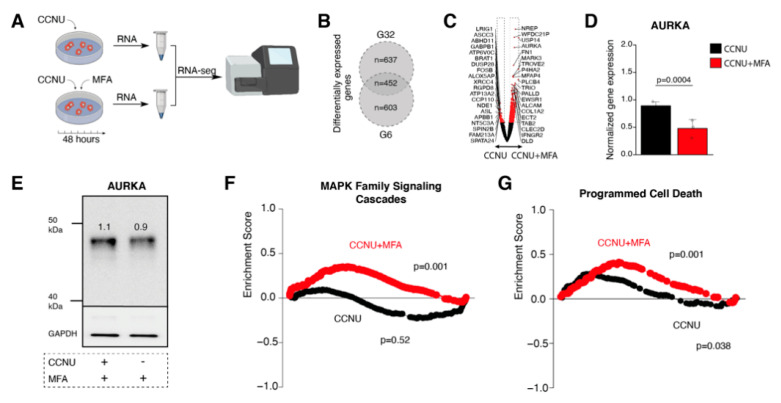
Meclofenamate-mediated sensitizing effects are accompanied by reduced Aurora kinase A expression and elevated programmed cell death pathway signaling. (**A**) Workflow illustration. After cells were treated with CCNU and the combination of CCNU and MFA for 48 h, RNA sequencing was performed. (**B**) Venn diagram depicting the number of differentially expressed genes of G6 and G32 cell populations separately and the number of shared differentially expressed genes. (**C**) Volcano plot of differentially expressed genes in CCNU monotreatment vs. combination treatment. (**D**) Barplot of AURKA expression. (**E**) Western blot analysis and densitometric quantification of AURKA protein expression. (**F**,**G**) Gene Set Enrichment Analysis of MSigDM REACTOME_MAPK_Family_Signaling_Cascades Pathway. (**F**) and REACTOME_Programmed_Cell_Death Pathway. (**G**) CCNU monotreatment compared to the untreated control is marked in black whereas CCNU monotreatment compared to combination treatment with MFA is marked in red. AURKA, Aurora kinase A; CCNU, lomustine; MFA, meclofenamate.

With regard to our findings of MFA to significantly increase the tumor cells’ susceptibility to lomustine therapy, we next analyzed gene expression changes for lomustine single to lomustine-MFA combination treatment. We found 637 differentially expressed genes in the G32 and 603 differentially expressed genes in the G6 cell population with 452 genes that were differentially expressed (CCNU vs. CCNU/MFA treatment) in both cell populations (Figure 6A–C). Aurora kinase A (AURKA) which is involved in the regulation of cell division and has been linked to glioblastoma proliferation capacity and therapy resistance [15], was among the most significant differentially expressed genes and showed a downregulation upon combination treatment (Figure 6C–E, Figures S2 and S3). A gene set enrichment analysis found MAPK signaling cascades (MSigDB REACTOME_MAPK_FAMILY_SIGNALING_CASCADES) as well as programmed cell death pathway signaling (MSigDB REACTOME_PROGRAMMED_CELL_DEATH) to be significantly upregulated for combination treatment (Figure 6E,F).

## 3. Discussion

The present study provides evidence that MFA as a gap junction inhibitor enhances glioblastoma cells’ vulnerability to lomustine-induced cell death independent of MGMT promoter methylation status. On the molecular level, this synergistic treatment effect was accompanied by reduced AURKA expression as well as elevated signaling of pathways involved in programmed cell death induction. 

Several studies have reported that inhibition of intercellular communication via gap junctions was accompanied by a sensitization for TMZ-mediated cell death [4,5,6,7]. With regard to TMZ as the standard chemotherapeutic agent in glioblastoma therapy, research on potential synergistic cytotoxic effects in the course of gap junction targeted therapies has focused on this alkylating drug, so far. However, with regard to the impact of lomustine in recurrent glioblastoma therapy as well as recent evidence for TMZ-lomustine combination to significantly improve survival in patients with newly diagnosed MGMT promoter methylated glioblastoma [9,11,16], there arises the question for the potential of a gap junction targeted therapeutic approach in the context of lomustine application. Our results show that while sensitizing effects for TMZ-induced toxicities were restricted to MGMT promoter methylated glioblastoma cells, lomustine revealed synergistic cytotoxic effects independent from MGMT promoter methylation status. Contrary to alkylating agent TMZ the cytotoxic effects of which mainly occur through alkylation of guanine residues [8], lomustine induces antitumoral effects beyond a mere DNA alkylation: Similar to other nitrosurea compounds lomustine leads to carbamoylation of amino acids [8] and exerts interstrand crosslinks [17], which in turn affect transcriptional, translational, and post-transcriptional molecular processes [11]. While the alkylating mechanisms of TMZ and lomustine are supposed to depend on the MGMT promoter methylation status, the abovementioned non-alkylating effects of lomustine might arise thereof independently. These differences in the mode of action might provide a rationale for our observation of a gap junction targeted therapy to synergize for lomustine-induced cytotoxic effects in both MGMT promoter methylated and unmethylated glioblastoma cells. Even though there is a lack of pharmacological studies, individual papers have reported lomustine plasma levels of about 2 µg/mL [18]. Taken the concentration of 20 µM (equivalent to 4.7 µg/mL) as chosen in the present manuscript, the present findings are in the near range of physiologically-measured concentrations especially with regard to in-vitro drug concentrations as a surrogate parameter for daily applicable doses and enhanced local tissue accumulation. However, the underlying mechanisms for a sensitizing effect of a gap junction-targeted therapy to cytotoxic medication is still far from understood. It has been speculated that the inhibition of intercellular cytosolic exchange might prevent the distribution of chemotherapeutics between interconnected tumor cells within the syncytial network culminating in toxic drug concentrations for the single tumor cell [3]. Furthermore, the disruption of intercellular survival signaling via heterotypic gap junction formations between glioblastoma cells and nonmalignant astrocytes might also add to the antitumoral effects of a gap junction-targeted therapeutic approach as it has been reported in mouse models of brain metastasizing breast and lung cancer [19]. 

When analyzing gene expression changes for lomustine single vs. lomusine-MFA combination therapy, we found AURKA among the most significant differentially expressed genes with a downregulation in case of combined drug application. As part of the serine/threonine kinase family, AURKA plays an important role in cell division through regulation of chromosome segregation [20]. In glioblastoma, AURKA has been linked to the tumor cells’ capacity of unlimited proliferation, maintenance of cellular stemness, and microinvasion [21,22,23,24]. Specific AURKA inhibitors have been reported to induce a G2/M phase arrest in glioblastoma cells culminating in increased percentage of apoptotic cells [25]. Notwithstanding the so-called “Cx43-interactome” that comprises the most prevalent protein-protein interaction networks for Cx43 on the base of functional enrichment analyses, reveales pivotal functions of gap junction-related signalings for cell cycle and mitotic processes among them microtubule-associated protein RP/EB family member 1 (MAPRE1) and cascein kinase isoform delta (CSNK1D) [26], further studies will be needed to unveil detailed molecular insights into the effects of gap junction inhibition on AURKA functioningThese assessments will have to clarify whether the observed downregulation of AURKA appears as a cellular mechanism of response to, or rather reflects, a direct molecular consequence of a gap junction-targeted therapy. 

Previous studies that had reported gap junction inhibition to render glioblastoma cells more vulnerable to TMZ-mediated cell death were mainly based on the application of experimental gap junction inhibitors that did not allow for a clinical translation [4,5,6,7]. In the present study, we made use of MFA as a FDA-approved NSAID that has been shown to inhibit gap junction-mediated intercellular cytosolic traffic in our primary glioblastoma cell culture system. Besides its clinical application, corroborative data also suggest MFA to sufficiently cross the blood–brain barrier: thus, systemic administration of MFA has been reported to exert CNS-mediated anticonvulsive effects in murine epilepsy models [27]. Therefore, MFA might constitute a clinically feasible drug to study for a potential survival benefit of a gap junction-targeted therapeutic approach in glioblastoma patients. However, up to date, there is a lack of studies sufficiently evaluating concentration-dependent toxicity of MFA. In a study of Koup, administration of 100 mg of MFA for 14 days every 8 h reached an average peak concentration of about 16 mM [28]. As these plasma levels measured in ten male volunteers are below the 50 mM of MFA chosen in the present study, further research will be needed in order to clarify both the effects of lower MFA concentrations in the setting of lomustine-induced antitumoral effects, and to explore the highest-feasible range of human in-vivo concentrations of MFA, without inducing toxic side effects.

## 4. Materials and Methods 

### 4.1. Cell Culture and Growth Conditions 

Glioblastoma cell populations (G6, G8, and G32) were purified from surgical specimens as previously described [29]. Resulting primary suspension cell populations were cultured in DMEM/F-12 medium supplemented with FGF (0.1 µg/mL), EGF (0.2 µg/mL), and B27 (1%). For experimental conditions, differentiation of suspension cells was provoked by letting them adhere in serum-enriched environment consisting of uncoated cell culture material in presence of DMEM enriched with 10% serum (FBS) and 1% Penicillin/Streptomycin. All cell populations were incubated at 37 °C in a water-saturated atmosphere containing 5% CO_2_. Differentiated cell populations were maintained for no more than twelve cell passages. Human sample preparation and analysis were approved by the local ethics committees of the University of Freiburg (protocol number 100020/09 and 472/15_160880) and the University of Ulm (protocol number 162/10). Written informed consent was obtained from all patients.

### 4.2. Characterization of Glioblastoma Cell Populations 

All cell populations were molecularly characterized by means of pyrosequencing with regard to existing MGMT promoter methylation and IDH1 and IDH2 mutation. DNA was extracted using the QIAamp DNA Mini Kit according to the recommendations of the manufacturer. Investigation of somatic mutations at codon R132 (IDH1) and R172 (IDH2) by pyrosequencing was performed as previously described [30]. For MGMT promoter methylation analysis DNA was subjected to bisulfite conversion and then analyzed as previously described [30]. Sequencing was performed on a Pyromark Q24 instrument. 

### 4.3. Viral Transduction 

For the detection of cells in fluorescence microscopy, primary glioblastoma cells were transduced with lentiviral particles Zsgreen (pZSgreen1-1 Vector, Clonetech). Therefore, suspension cell populations were seeded with a density of 5 × 10^4^ cells/mL in a laminin-coated petri dish and cultivated in medium enriched with polybrene (1 µL/mL) and virus particles (1 µL/mL) for 24 h. Efficacy of transduction was measured 24 h after the lentiviral particles were discarded. 

### 4.4. Reagents 

Meclofenamate and lomustine were purchased from Sigma. For preparation of 100-mM stock solutions, meclofenamate was dissolved in dimethyl sulfoxide (DMSO) and lomustine in ethanol. Both drugs were aliquoted and stored at −20 °C. Final DMSO and ethanol concentrations did not exceed 0.1%. 

### 4.5. Immunofluorescence

Immunostaining was performed as previously described [4]. Briefly, cells were seeded on glass coverslips coated with laminin. Then, cells were fixed with 4% paraformaldehyde, permeabilized with 20% methanol followed by incubation with a primary antibody mix of Cx43 and ß-tubulin and the corresponding secondary antibodies. In addition, the cell nuclei were stained with DAPI Fluoromount mounting medium. The immunofluorescence images were acquired with an AX70 microscope from Zeiss and evaluated with the Zeiss Zen Blue software. 

### 4.6. Protein Immunoblotting 

For Western blot analysis, 50 µg protein of each sample were separated electrophoretically in SDS-polyacrylamide gels and transferred to nitrocellulose membranes as previously described [31]. The nitrocellulose membranes were then incubated overnight at 4 °C with the specified primary antibodies. After washing for several times, the corresponding secondary antibody was added and the membranes were incubated for another hour. After several washes, the chemiluminescent substrate was added to the membranes and the signals were visualized with the ChemoCam Imager (Intas). 

### 4.7. Calcein Dye Transfer

To investigate the redistribution of calcein fluorescent dye, unstained “acceptor” cells were seeded and incubated for 24 h. Then, “donor” cells were incubated for 20 min in growth medium containing 5 µM of calcein AM. Hereafter, medium of the previously seeded unstained cells was replaced to contain the calcein-stained fluorescent “donor” cells (ratio of 1:4) and various concentrations of meclofenamate. Fluorescence images were taken using The IncuCyte© S3 Live-Cell Analysis System hourly for 6 h. Subsequent image processing and analysis was performed using a personalized CellProfiler pipeline [32] and calcein diffusion rate was calculated as: number of acceptor cells/number of donor cells.

### 4.8. MTT

The metabolic activity of the treated glioblastoma cells was investigated using a MTT assay as previously described [4]. Therefore, cells were treated with ascending concentrations of lomustine and meclofenamate for 72 and 144 h. Then, one tenth of the MTT stock solution (1 mg/mL) was added to the cells which were incubated for another 3 h. The absorbance at 550 nm was determined with the µQuant spectrometer. 

### 4.9. Proliferation Measurement

For assessment of cell proliferation, cell treatment was performed and subsequently, fluorescence images were taken using The IncuCyte© S3 Live-Cell Analysis System in a 24-h interval for 144 h. The obtained data were analyzed using the device-specific software Incucyte 2019B to precisely determine the number of cells. 

### 4.10. Flow Cytometric Analysis of Cell Death

To analyze cell death after treatment with lomustine, meclofenamate or the combination of both, DNA-fragmentation of propidium iodide-stained nuclei was assessed by flow cytometric analysis using a FACSCanto II device, as previously described [29]. Dead cells (irrespective of the form of cell death that has been passed through (apoptosis, necroptosis, necrosis or autophagy)) are characterized by DNA fragmentation and loss of nuclear DNA content [33]. As a consequence, dead cells treated with Triton-X and stained with PI exhibit a hypodiploid (sub-G_1_) peak, which can easily be distinguished from the narrow peak of cells with normal (diploid) DNA content [33]. For this purpose, adherent cells were enzymatically detached after 144 h of treatment with trypsin (0.1 %), centrifuged for 5 min at 1300 rpm, and washed twice in PBS. Obtained pellets were then resuspended in FACS buffer consisting of trisodium citrate dihydrate (0.05 %), Triton-X (0.05 %), and propidium iodide (0.05 mg/mL). Cells were then incubated for 1 h at 4 °C with exclusion of light. Acquired FCS files were analyzed using the FlowJo software version 10.4 and specific DNA fragmentation rates were calculated as follows: 100 × (experimental DNA fragmentation (%) − spontaneous DNA fragmentation (%))/(100% − spontaneous DNA fragmentation (%)).

### 4.11. Bliss Independence Analysis 

The Bliss independence model was used to investigate the combination effect of lomustine and meclofenamate using the flow cytometric data on cell death. The quotient between the arithmetically predicted response for combination drug treatment and experimentally observed response was defined as Bliss value. The predicted response for combined treatment was calculated as: E = (a + b) − (a × b), where a and b were the DNA fragmentation rates of lomustine (a) and meclofenamate (b) at a given concentration. A synergistic interaction was defined as Bliss value >1.1. 

### 4.12. RNA Sequencing 

After cells were cultivated for 48 h, purification of RNA was achieved using the RNeasy Mini Kit (Qiagen). The reverse transcription reaction was performed using the Maxima H Minus Reverse Transcriptase. For preparation of RNA sequencing, the PCR Barcoding Kit and cDNA-PCR Sequencing Kit (Oxford Nanopore Technologies) were utilized as recommended by the manufacturer. RNA sequencing was performed using the MinION Sequencing Device, the SpotON Flow Cell and MinKNOW software (Oxford Nanopore Technologies) according to the manufacturer’s instructions. Base calling was performed by Albacore implemented in the nanopore software. Only D2-reads with a quality score above 8 were used for further alignment. Reads were re-arranged in accordance to their barcode and trimmed by Porechop (https://github.com/rrwick/Porechop (accessed on 15 December 2020)). Alignment was performed by minimap2 (https://github.com/lh3/minimap2 (accessed on 15 December 2020)) and processed by sam-tools. Mapped reads were normalized by DESeq2. The expression matrix was analyzed with AutoPipe (https://github.com/heilandd/AutoPipe (accessed on 15 December 2020)) by a supervised machine-learning algorithm. Visualization was performed using VisLab Expression Data Viewer (https://github.com/heilandd/Vis_Lab1.5 (accessed on 15 December 2020)). 

## 5. Conclusions

The present study is the first to show that inhibition of intercellular communication via gap junctions sensitizes primary glioblastoma cells to lomustine-mediated toxicity. This effect was independent of MGMT promoter methylation status and showed reduced AURKA expression and elevated programmed cell death pathway signaling. With regard to MFA as a clinically-approved NSAID to be drug-repurposed as a gap junction inhibitor, these findings support the concept of a gap junction-targeted therapy to constitute a clinically-feasible way to further reinforce lomustine-induced antitumoral effects. 

## Data Availability

All data generated or analysed during this study are included in this published article (and its Supplementary Information Files).

## Supplementary Materials

The following are available online at https://www.mdpi.com/1424-8247/14/3/195/s1, Figure S1: Determination of effective drug concentrations; Figure S2: Top 20 upregulated (red) and downregulated (blue) genes for combination treatment of CCNU and MFA. CCNU, lomustine; MFA, meclofenamate. Figure S3: Western blot analysis for AURKA and Cx43 protein expression. AURKA, Aurora kinase A; CCNU, lomustine; Cx43, connexin 43; MFA, meclofenamate.
