# Supplementary material for: Inhibition of Intercellular Cytosolic Traffic via Gap Junctions Reinforces Lomustine-Induced Toxicity in Glioblastoma Independent of MGMT Promoter Methylation Status
Source: Pharmaceuticals (Basel). 2021 Feb 27;14(3):195. doi: 10.3390/ph14030195 (PMC7997332; doi:10.3390/ph14030195)
Supplement: Supplementary file 1 [file pharmaceuticals-14-00195-s001.pdf]

pharmaceuticals

Supplementary Materials

Inhibition of intercellular cytosolic traffic via gap junctions reinforces lomustine-induced toxicity in glioblastoma independent of MGMT promoter methylation status

Matthias Schneider, Anna-Laura Potthoff, Bernd O. Evert, Marius Dicks, Denise Ehrentraut, Andreas Dolf, Elena N. C. Schmidt, Niklas Schäfer, Valeri Borger, Torsten Pietsch, Mike-Andrew Westhoff, Erdem Güresir, Andreas Waha, Hartmut Vatter, Dieter H. Heiland, Patrick Schuss, Ulrich Herrlinger

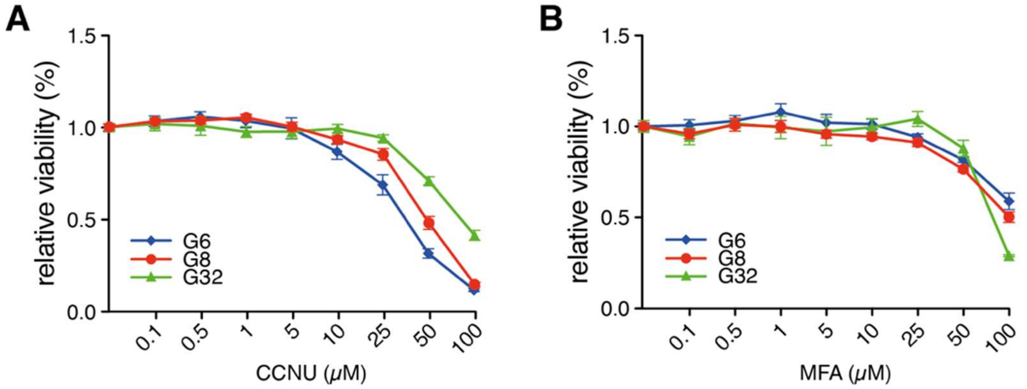

Figure S1. Determination of effective drug concentrations. Relative cell viability was assessed 144 hours after treatment with various concentrations of CCNU (left) and MFA (right) for G6, G8 and G32 cell populations. Untreated controls were defined as 1. Mean ± SEM is depicted. CCNU, lomustine; MFA, meclofenamate.

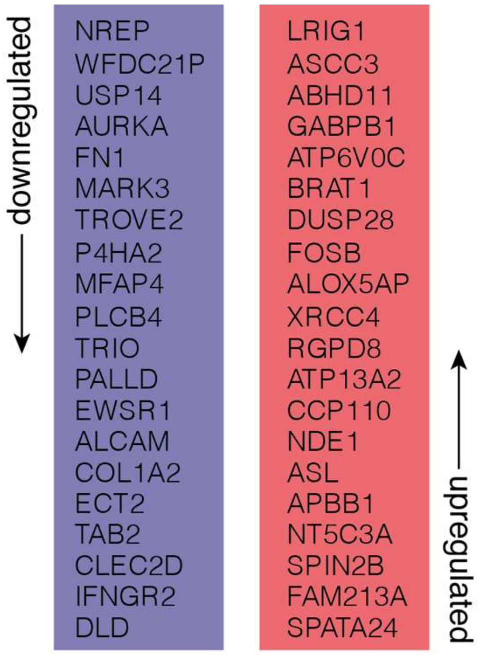

Figure S2. Top 20 upregulated (red) and downregulated (blue) genes for combination treatment of CCNU and MFA. CCNU, lomustine; MFA, meclofenamate.

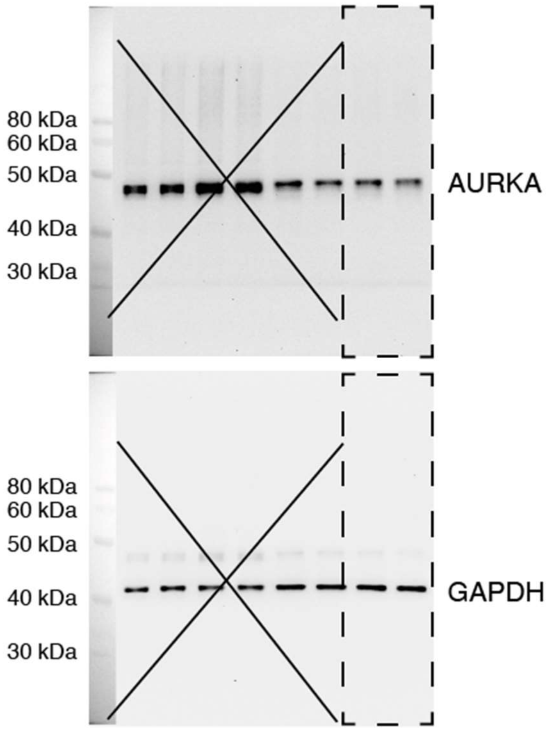

Figure S3. Western blot analysis for AURKA and Cx43 protein expression. AURKA, Aurora kinase A; CCNU, lomustine; Cx43, connexin 43; MFA, meclofenamate.
